# Validity of a simple footprint assessment board for diagnosing the severity of flatfoot: a prospective cohort study

**DOI:** 10.1186/s12891-021-04154-3

**Published:** 2021-03-18

**Authors:** Seikai Toyooka, Naoya Shimazaki, Youichi Yasui, Shuji Ando, Yasuaki Saho, Takumi Nakagawa, Hirotaka Kawano, Wataru Miyamoto

**Affiliations:** 1 Department of Orthopaedic Surgery, Teikyo University School of Medicine, Tokyo, Japan; 2 Department of Orthopaedic Surgery, Shimazaki Hospital, Ibaraki, Japan; 3 Department of Information Engineering, Tokyo University of Science, Tokyo, Japan; 4 Faculty of Medical Technology, Teikyo University Institute of Sports Science and Medicine, Tokyo, Japan

**Keywords:** Flatfoot, Footprint assessment board, Arch height, Navicular index

## Abstract

**Background:**

A simple, non-quantitative, and cost-effective diagnostic tool would enable the diagnosis of flatfoot without need for specialized training. A simple footprint assessment board that investigates which toe the cord passes through from the centre point of the heel to the most lateral point of the medial contour of the footprint has been developed to assess flatfoot. The purpose of this study was to verify the validity of a simple footprint assessment board for flatfoot.

**Methods:**

Thirty-five consecutive patients with foot pain, foot injury, or any associated symptoms who underwent computed tomography (CT) were analysed prospectively. At the time of the CT scan, a footprint analysis using a simple footprint assessment board was performed. The navicular index, tibiocalcaneal angle, and calcaneal inclination angle were evaluated by CT to assess flat feet. These three criteria were compared to those evaluated with the simple footprint assessment board by regression analysis. In addition, the same analysis was conducted separately for young, middle-aged, and older patients in order to investigate each age group.

**Results:**

The navicular index and tibiocalcaneal angle generally decreased as the score of the simple footprint assessment board increased. Calcaneal inclination angle generally increased as the score of the simple footprint assessment board increased. As the scores of the simple footprint assessment board decreased by approaching the great toe, the navicular index and tibiocalcaneal angle were higher and calcaneal inclination angle was lower, which is indicative of a higher likelihood of flatfoot. The scores derived from the simple footprint assessment board was correlated with these three criteria measured by CT, not only when the result of simple footprint assessment board was set as a non-continuous variable but also when the result was set as a continuous variable. The results of the age-stratified survey were similar for all groups.

**Conclusions:**

The findings of this study suggest that a simple footprint assessment board can be potentially useful to detect flatfoot.

**Trial registration:**

Retrospectively registered.

**Supplementary Information:**

The online version contains supplementary material available at 10.1186/s12891-021-04154-3.

## Background

Flatfoot deformity is a medical condition characterized by a flattened arch on the medial border of the plantar foot wherein the entire sole of the foot comes into near-complete contact with the ground [1]. The prevalence of flatfoot has been reported as approximately 26.5% [2, 3]. A compromised function of the foot arc may increase the risk of overuse injury and continuous pain, the former of which can cause advanced hindfoot deformity such as osteoarthritis of the subtalar and Chopart joints in patients with flatfoot [4, 5]. In addition, flatfoot is also associated with osteoarthritis of the knee and hip dysplasia, and early diagnosis and treatment are crucial for the prevention of disease progression [6, 7].

Several clinical diagnostic approaches have been adopted to identify flatfoot, including the assessment of clinical symptoms [8, 9], radiographic imaging [8, 10], and footprint analysis [11–14]. The most common diagnostic measure for flatfoot is the assessment of clinical symptoms and physical findings; however, the processes of evaluation can be subjective and may require clinical experience [15]. In the case of radiographic diagnosis, a set of angular parameters is used to assess the degree of deformity from standard dorsoplantar and lateral radiographs of the weight-bearing feet [1]. There are several disadvantages associated with radiological assessment, including the difficulty in determining these angles, discrepancies in imaging quality due to varying competencies of radiologic technicians, inter- or intraobserver error, and exposure to radiation [8, 16–18].

On the other hand, footprint analysis is a simple, quick, cost-effective, and readily available method and has been recommended as a screening tool for flatfoot [11–14]. Although previous studies have developed various footprint analyses for the assessment of the arch that have been considered reliable by many researchers, these procedures require measurements of area, angle, and distance using an image of the footprint, in addition to occasional calculations to determine the ratio of the distances [11, 13, 14, 16]. A simplified and non-quantitative diagnostic tool would be greatly beneficial for medical workers to diagnose flatfoot without need for specialized training. A medical equipment manufacturer has recently developed a simple footprint assessment board that investigates which toe the cord passes through from the centre point of the heel to the most lateral point of the medial contour of the footprint with a thermochromic surface to describe the footprint and assess flatfoot; however, there has been no verification on the diagnostic accuracy obtained by this board. The purpose of this study was to verify the validity of a simple footprint assessment board for flatfoot. The hypothesis was that there is a correlation between the results of the simple assessment board and the radiological assessment for the diagnosis of flatfoot. If the hypothesis can be proven, a simple tool can be used for an accurate assessment of flatfoot without radiation exposure, high cost, and time-consuming measurements. 

## Methods

### Patients and design

Data for consecutive patients with foot pain, foot injury, or any associated symptoms who underwent computed tomography (CT) between January 2019 and June 2020 at a single institution were analysed prospectively. At the time of the CT scan, a footprint analysis using a simple footprint assessment board (Arch Check Board, NIPPON SIGMAX, Tokyo, Japan) was also performed. The results for the arch height evaluated by CT was compared to those evaluated by the simple footprint assessment board. The study protocol was approved by the institutional review board of the author’s institution and all patients provided informed consent. For patients under 18 years of age, informed consent was granted by their parents or legally authorized representatives. The exclusion criteria were as follows: history of lower extremity surgery, patients with symptoms that prevent them from loading their lower extremities, patients with a capillary refill time of more than 2 s who were not expected to produce a clear thermal impression on the thermochromic surface of the simple footprint assessment board.

### Measurements

**Fig. 1:**
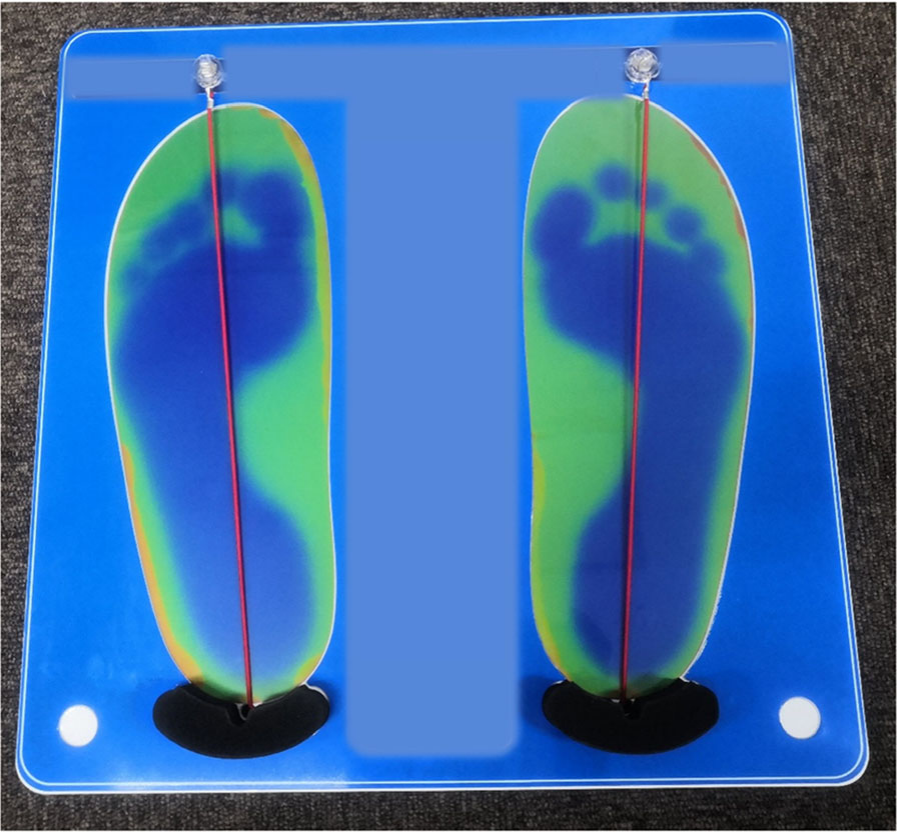
A photograph of the simple footprint assessment board with thermochromic discoloration of footprint

**Fig. 2:**
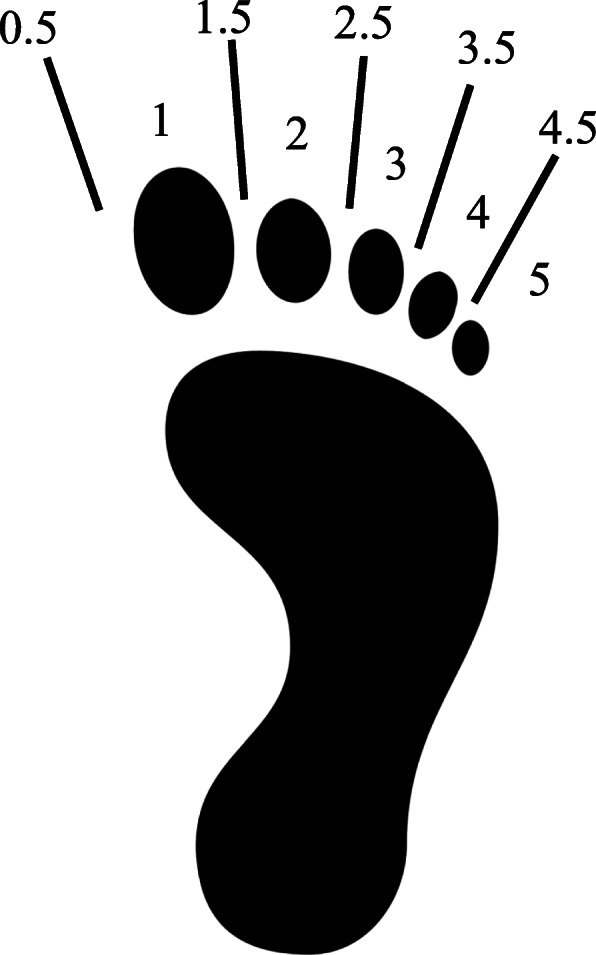
Scoring for the simple footprint assessment board

For each person included in this study, anthropometric variables (age, gender, and body mass index) were examined, in addition to the reason for undergoing CT. In this study, the validity to assess arch height was evaluated with a simple footprint assessment board. Patients placed their feet on a thermochromic sheet that was placed on the top of this board to check for discoloration. The discoloration produced an accurate footprint on the board. A cord was attached to the board which was fixed to the centre point of the heel with a magnet at its other end. To measure the arch height, the cord was set up to contact the most lateral point of the medial contour of the footprint and fixed to a magnetic strip located distally to the toes (Fig. 1). The evaluation was performed by determining which toe the cord passed through and scored as follows: 1, the cord passed through the footprint of the great toe; 2, through the second toe; 3, through the third toe; 4, through the fourth toe; 5, through the fifth toe. When the cord passed through the medial region of the great toe, the evaluation was defined as 0.5, and when the cord passed between toes, a score of 0.5 was added to the score of lesser toes (i.e., 1.5 when the cord passed between the great toe and second toe, and 2.5 when passed between second and third toes) (Fig. 2). This measurement was performed by a skilled radiologic technician who was blinded to the patient’s background. A greater score indicated a greater arch height.

**Fig. 3:**
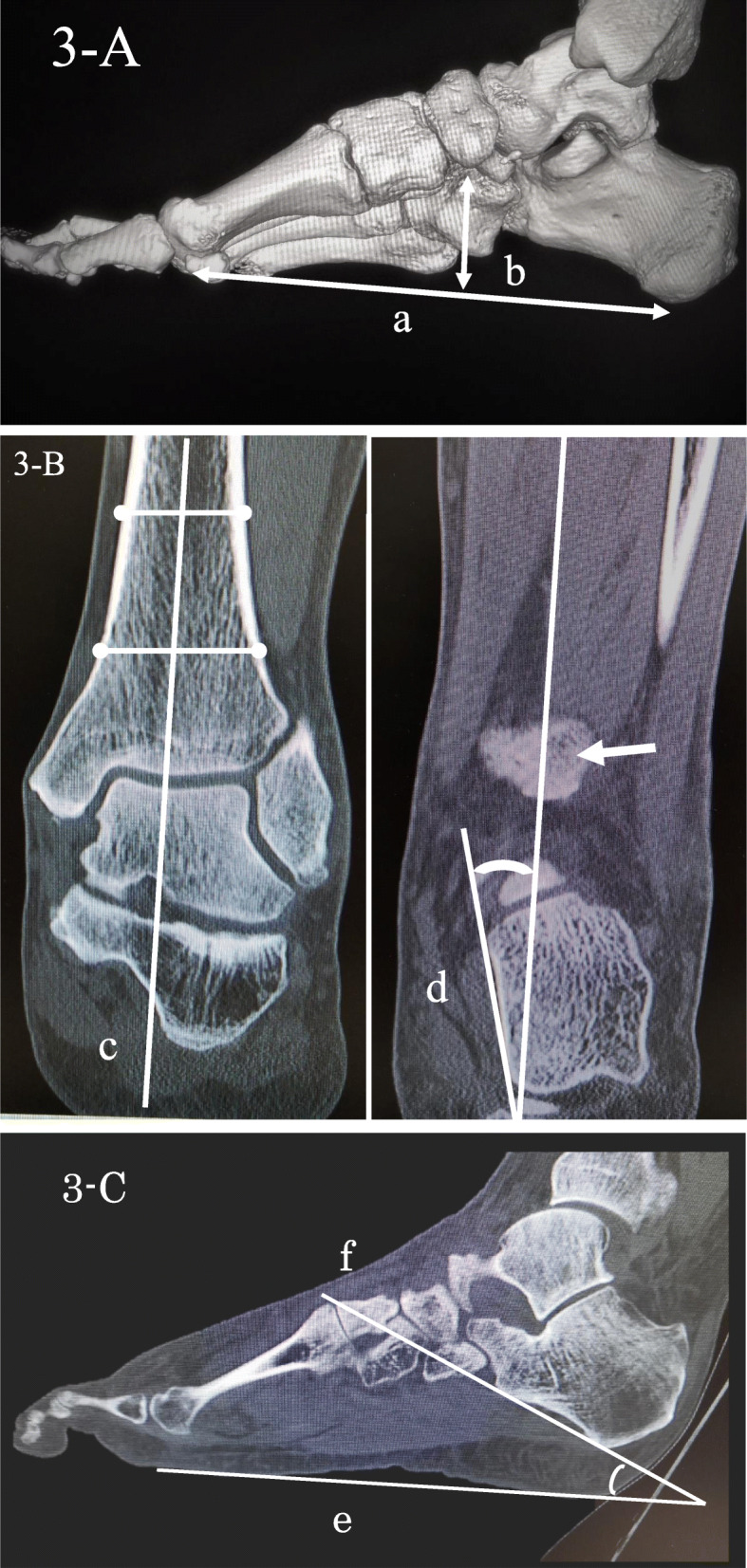
The methodology of measuring the longitudinal arch length and navicular height with 3DCT imaging (A), tibiocalcaneal angle with coronal CT image (B), and calcaneal inclination angle with sagittal CT image (C). A. a: The length of the longitudinal arch. b: The navicular height. B. c: The axis of the distal tibia. d: A line of the medial calcaneal contour. White arrow: The posterior tibial cortex. C. e: The plantar line. f: The most inferior point of the calcaneal tuberosity and the inferior edge of the anterior process of the calcaneus

CT (Toshiba Aquilion, Canon Medical Systems Cooperation, Otawara, Japan) was performed with the standard bone CT protocol with 0.5-mm axial sections in three planes, with a tube voltage of 120 kV. After creating a 3D computed tomography image, three criteria were evaluated. First, the navicular index was evaluated according to a method described by Roth et al. [19]. They introduced the navicular index as a new measure to distinguish between flatfoot and normal foot. A greater navicular index suggested a higher likelihood of a flatfoot. A line connecting the lowest point of the first metatarsal head to the lowest point of the calcaneus was created with the 3D CT image. The distance between the lowest point of the first metatarsal head and the lowest point of the calcaneus was defined as “the length of the longitudinal arch.” A plane was subsequently created to passed through the lowest point of the first metatarsal head, the lowest part of the fifth metatarsal head, and the lowest part of the calcaneus. The distance of the perpendicular line from the lowest point of the navicular bone to this plane was measured and defined as “the navicular height” (Fig. 3A). The navicular index which was calculated by dividing the length of the longitudinal arch with navicular height was investigated. A higher navicular index indicated a lower arch height. Second, the tibiocalcaneal angle was evaluated according to a method described by Lee et al. [20]. This angle was defined as the angle between the axis of distal tibial shaft and the medial calcaneal contour. The axis of the distal tibial shaft was first drawn through the centre of the tibial shaft, which was defined by the midpoint of two pairs of points on the distal tibial cortex in coronal CT image through the maximum width of the tibial shaft. Then, a line of the medial calcaneal contour was drawn along the medial calcaneal wall in coronal CT image through the posterior tibial cortex. The resulting angle between these 2 lines was defined as the tibiocalcaneal angle (Fig. 3B). Third, the calcaneal inclination angle was evaluated according to a method described by de Cesar Netto et al. [21]. This angle was defined as the junction of 1) the plantar line and 2) a line connecting the most inferior point and edge of the calcaneal tuberosity and anterior process of the calcaneus in sagittal CT image, respectively (Fig. 3C). The latter two are both part of the assessment used to evaluate flat feet. Since these two radiographic angles are well correlated with weight-bearing and non-weight-bearing conditions [21, 22], the measurement can be carried out without concern for load conditions. SYNAPSE VINCENT Ver. 3.3 (FUJIFILM Cooperation, Tokyo, Japan) was used for these measurements as an image analysis software. CT images were evaluated independently by orthopaedic surgeons with 15 years of clinical experience and were blinded to the clinical and patient data.

The relationship between the results of evaluation by the simple footprint assessment board and the navicular index, tibiocalcaneal angle, and calcaneal inclination angle by CT was evaluated. In order to examine the results of the survey that were stratified by age, the same survey was conducted for the young group (≤ 35 years), the middle-aged group (36–55 years), and the older group (> 56 years).

Intrarater reliability in the measurement of the simple footprint assessment board was assessed using the intraclass correlation coefficient (ICC). Measurements were repeated two times on every foot in this study. In order to evaluate the reliability of radiographic variables, intraobserver and interobserver reliabilities were assessed using ICC. Measurements of the navicular index, tibiocalcaneal angle, and calcaneal inclination angle by CT were repeated two times on every patient for intraobserver reliability. To evaluate for interobserver reliability, another orthopaedic surgeon conducted the measurements and comparisons for all patients.

### Statistical analysis

When examining the relationship between the result of the simple footprint assessment board and the navicular index, tibiocalcaneal angle, and calcaneal inclination angle, the current study was analysed using dummy variables because the spacing of the toes was not consistent for each individual and was not a continuous variable [23]. The results of the simple footprint assessment board were set as explanatory variables and the navicular index, tibiocalcaneal angle, and calcaneal inclination angle, were set as objective variables for the analysis. With the medial side of the first toe as a reference, a dummy variable was created as the results of the simple footprint assessment board (1 if applicable, 0 otherwise). Then, a regression analysis was conducted with all dummy variables as explanatory variables to evaluate how well the navicular index could be explained or predicted from the scores of the simple footprint assessment board. In addition to this, a regression analysis was conducted with the scores of the simple footprint as a continuous variable. The ICC was calculated using SPSS version 12 software (SPSS Inc., Chicago, IL).

The number of cases were difficult to set by power analysis in this test due to the use of dummy variables, and a statistician was consulted prior to determining the number of cases. The number of cases that could be secured for each assessment board value for arch height (divided into 8 levels between the medial side of the first toe to the fourth toe) was set as over 32, which equated to approximately 4 cases each.

## Results

The current study examined 35 ft of 30 patients with a mean age of 44.7 years. The patient characteristics are shown in Table 1. This included two patients who had already been diagnosed with flatfoot from clinical findings.

**Table 1:** Patient characteristics

n	35 (30 patients)
Mean age	44.7 (14–85)
Sex	Male, 21; Female, 9
Affected side	Right, 20; Left, 15
Mean height (cm)	163.8 ± 9.5
Mean weight (kg)	62.5 ± 14.7
Mean BMI	23.2 ± 5.1
Reason for undergoing CT	Foot injury: 14 Foot pain: 5 Hallux valgus: 4 Lisfranc osteoarthritis: 4 Accessory navicular: 3 Flat foot: 2, Ankle sprain: 1, Plantar fasciitis: 1 Sesamoid bones: 1

In the navicular index, regression analysis with the scores of the simple footprint assessment board as a dummy variable showed that when a footprint assessment board score of 0.5 was set as the reference point, the regression coefficients generally decreased as the simple footprint assessment board score increased (Table 2). Although the coefficient factor for a score of 2.5 was greater than that of 2, the rest of the results showed that the navicular index decreased as the scores of the simple footprint assessment board increased. As the scores of the simple footprint assessment board decreased by approaching the great toe, the navicular index was higher, which is indicative of a higher likelihood of flatfoot. In addition, regression analysis with the value of the simple footprint assessment board as a continuous variable showed that there was a significant correlation between the score of simple footprint assessment board and navicular index: y = − 0.883x + 6.505, *p* < 0.001.

**Table 2:** Navicular index. Regression analysis with the value of the simple footprint assessment board as a dummy variable

Score of arch check board	n	Mean navicular index	Regression factor	Standard error	t-value	*P*-value	Coefficient of determination	Adjusted coefficient of determination
(Intercept)			6.370	0.479	13.299	0.000	0.658	0.569
Score 0.5 (Reference)	2	6.4						
Score 1	5	6.0	−0.382	0.567	−0.674	0.506		
Score 1.5	5	5.2	−1.200	0.567	−2.117	0.044		
Score 2	9	4.4	−1.974	0.530	−3.729	0.001		
Score 2.5	6	4.5	−1.905	0.553	−3.444	0.002		
Score 3	6	3.9	−2.465	0.553	−4.457	0.000		
Score 3.5	1	3.7	−2.670	0.830	−3.218	0.003		
Score 4	1	3.0	−3.370	0.830	−4.062	0.000		
Score of arch check board	Regression factor		Standard error		t-value	*P*-value	Coefficient of determination	Adjusted coefficient of determination
(Intercept)	6.505		0.277		23.470	0.000	0.602	0.590
Score	−0.883		0.125		−7.060	0.000		

Score of arch check board (explanatory variables: x)

Navicular index (objective variables: y)

Regression analysis with the value of the simple footprint assessment board as a continuous variable

Score of arch check board (explanatory variables: x)

Navicular index (objective variables: y),

In the tibiocalcaneal angle, regression analysis with the scores of the simple footprint assessment board as a dummy variable showed that when a footprint assessment board score of 0.5 was set as the reference point, the regression coefficients generally decreased as the simple footprint assessment board score increased (Table 3). In addition, regression analysis with the value of the simple footprint assessment board as a continuous variable showed that there was a significant correlation between the score of simple footprint assessment board and navicular index: y = − 4.200x + 24.064, *p* < 0.001.

**Table 3:** Tibiocalcaneal angle. Regression analysis with the value of the simple footprint assessment board as a dummy variable

Score of arch check board	n	Mean navicular index	Regression factor	Standard error	t-value	*P*-value	Coefficient of determination	Adjusted coefficient of determination
(Intercept)			26.000	1.232	21.107	0.000	0.876	0.838
Score 0.5 (Reference)	2	6.4						
Score 1	5	6.0	−5.940	1.458	−4.075	0.000		
Score 1.5	5	5.2	−10.440	1.458	−7.163	0.000		
Score 2	9	4.4	−10.917	1.422	−7.675	0.000		
Score 2.5	6	4.5	−12.520	1.458	−8.590	0.000		
Score 3	6	3.9	−13.900	1.422	−9.772	0.000		
Score 3.5	1	3.7	−16.300	2.134	−7.640	0.000		
Score 4	1	3.0	−17.000	2.134	−7.968	0.000		
Score of arch check board	Regression factor		Standard error	t-value	*P*-value	Coefficient of determination	Adjusted coefficient of determination	
(Intercept)	24.064		0.977	24.630	0.000	0.760	0.752	
Score	−4.200		0.438	−9.590	0.000			

Score of arch check board (explanatory variables: x)

Tibiocalcaneal angle (objective variables: y)

Regression analysis with the value of the simple footprint assessment board as a continuous variable

Score of arch check board (explanatory variables: x)

Tibiocalcaneal angle (objective variables: y),

In the calcaneal inclination angle, regression analysis with the scores of the simple footprint assessment board as a dummy variable showed that when a footprint assessment board score of 0.5 was set as the reference point, the regression coefficients generally increased as the simple footprint assessment board score increased (Table 4). In addition, regression analysis with the value of the simple footprint assessment board as a continuous variable showed that there was a significant correlation between the score of simple footprint assessment board and navicular index: y = 6.053x + 12.069, *p* < 0.001.

**Table 4:** Calcaneal inclination angle. Regression analysis with the value of the simple footprint assessment board as a dummy variable

Score of arch check board	n	Mean navicular index	Regression factor	Standard error	t-value	*P*-value	Coefficient of determination	Adjusted coefficient of determination
(Intercept)			14.500	1.918	7.560	0.000	0.843	0.795
Score 0.5 (Reference)	2	6.4						
Score 1	5	6.0	3.580	2.269	1.578	0.128		
Score 1.5	5	5.2	8.020	2.269	3.534	0.002		
Score 2	9	4.4	8.667	2.215	3.913	0.001		
Score 2.5	6	4.5	13.000	2.269	5.729	0.000		
Score 3	6	3.9	15.167	2.215	6.849	0.000		
Score 3.5	1	3.7	20.500	3.322	6.171	0.000		
Score 4	1	3.0	22.500	3.322	6.773	0.000		
Score of arch check board	Regression factor		Standard error		t-value	*P*-value	Coefficient of determination	Adjusted coefficient of determination
(Intercept)	12.069		1.165		10.360	0.000	0.822	0.816
Score	6.053		0.522		11.590	0.000		

Score of arch check board (explanatory variables: x)

Calcaneal inclination angle (objective variables: y)

Regression analysis with the value of the simple footprint assessment board as a continuous variable

Score of arch check board (explanatory variables: x)

Calcaneal inclination angle (objective variables: y),

The results of the age-stratified survey were the same as the overall results for all groups (Additional file 1).

ICC of the simple footprint assessment board based on the data of every foot in this study was 0.93. The interobserver ICC of the navicular index, tibiocalcaneal angle, and calcaneal inclination angle were 0.99, 0.92, and 0.93 respectively. The intraobserver ICC of the navicular index, tibiocalcaneal angle, and calcaneal inclination angle were 0.89, 0.94, and 0.89 respectively.

## Discussion

Our results clearly indicate that arch height which was evaluated by the simple footprint assessment board was correlated with the navicular index, tibiocalcaneal angle, and calcaneal inclination angle by CT. The same results were not only obtained for all ages simultaneously, but also for young, middle-aged, and older patients. This means that simple footprint assessment board can potentially be a substitute to CT for the diagnosis of flatfoot for all patients.

Various footprint-based analyses for foot arch assessment have been developed in previous studies. A previous report by Cavanagh and Rodgers measured and calculated the arch index as defined as the proportion of area for the middle third and total toeless footprint [24]. Other reports have described the use of Irwin’s footprint index or similar modified approaches to determine the severity of flatfoot by calculating the area of the arch in a footprint [12, 25, 26]. Another common assessment is the use of Clarke’s angle, which is calculated by the angle between 1) the medial tangential line joining the medial margin of the first metatarsal head/heel, and 2) the line joining the first metatarsal head and apex of the concavity in the medial longitudinal arch [12, 14, 27, 28]. Forriol and Pascual described the use of Chippaux-Smirak index to determine foot arch development, which was calculated by the ratio of the maximum width of the metatarsals to the minimum width of the arch [12, 14, 28–31]. In addition, Staheli et al. developed an index for plantar arch as defined by the ratio of the midfoot- to hindfoot-width that is used as an indicator of foot arch development [12, 14, 31, 32]. Many researchers have recommended these procedures for foot arch assessment as a reliable screening method [12, 13]. These procedures are simple and do not require any special equipment; however, the diagnostic methods require difficult and time-consuming tasks such as the measurement of area, angle, distance in addition to the calculation of their ratio [14].

Because flatfoot is a common disorder, a simpler and quicker diagnostic tool for flatfoot may be useful for a more diverse range of medical and healthcare professionals. The diagnosis of the disorder has to be dealt with across a spectrum of practitioners that is not limited to orthopaedic surgeons but also those who do not normally perform radiographic examinations, such as family practitioners, non-physicians, physical therapists, athletic trainers, orthotic prosthetists, and shoemakers. In this study, the effectiveness of a simple footprint assessment board that investigates which toe the cord passes through from the centre point of the heel to the most lateral point of the medial contour of the footprint with a thermochromic surface was evaluated. This board features the ability to accurately reproduce a footprint by the discoloration of its surface according to the patient’s foot temperature, and the degree of flatfoot can be examined using the image of the footprint on the board by checking which toe the cord passes through from the centre point of the heel to the most lateral point of the medial contour of the footprint. Traditionally, a pedograph has been used for footprint analysis. This device consists of an inked rubber membrane of small grid lines that are imprinted on an underlying sheet of paper when a foot passes over it. In contrast, the simple footprint assessment board can be repeatedly used without ink or paper.

As a result of this study, the scores of the simple footprint assessment board was correlated with the navicular index, tibiocalcaneal angle, and calcaneal inclination angle measured by CT, not only when the result of the simple footprint assessment board was set as a non-continuous variable but also when the result was set as a continuous variable. The intrarater reliability of the simple footprint assessment board, which was measured twice on every foot in this study, was high. Therefore, the data obtained from this simple footprint assessment board proved to be reproducible and reliable.

In the present study, one of the criteria which we used was the navicular index reported by Roth et al. [19]. They reported that values of the navicular index for flatfoot were in the interval from 4.75 to 31.20 (median 8.98) and for normal-arched foot 3.58 to 22.6 (median 5.48). Two of 35 ft had already been diagnosed as flatfoot based on clinical findings. The navicular index values of these 2 ft were 7.32 and 6.76, and the scores in the simple footprint assessment board were 0.5 and 1, respectively. On the other hand, it has been reported that if calcaneal inclination angle is less than 18 degrees, flat feet are more likely [33]. These results for known cases of flatfoot suggest that the diagnosis of flatfoot is highly likely if the cord of the simple footprint assessment board either passes through the great toe or over its medial side.

The height of the navicular bone was assessed using CT images instead of radiographic images in this study. The measurements of various angles on radiographs are always challenging due to superimposition of the bones. Furthermore, radiographs lack reproducibility and are associated with rotational and fan distortions [18]. In contrast, CT images have the advantage of multiplanar capabilities and higher resolutions. Since the interobserver and intraobserver ICC were sufficiently high, the evaluation of radiographic variables was reliable.

This study has limitations. Firstly, CT images were taken in the supine position and did not undergo imaging under load. Compared with non-weight-bearing images, weight-bearing images better demonstrate the severity of osseous derangement in patients with flat foot [21]. The results of the present study can show the usefulness of the simple footprint assessment board to diagnose rigid flat foot which is a loss of medial arch in an unloaded condition. On the other hand, the validity of this board for the diagnosis of flexible flatfoot, which is loss of the inner arch in a loaded condition and more common in children, could not be examined because non weight-bearing CT images were applied to measure the navicular index. However, the results of the tibiocalcaneal angle and calcaneal inclination angle were well-correlated with both weight-bearing and non-weight-bearing conditions [21, 33]. Future research should be conducted with weight-bearing CT images for radiological evaluation to clarify the usefulness of simple tools like the simple footprint assessment board for diagnosis of flexible flatfoot. Secondly, all included patients suffered from foot pain, foot injury, or symptoms around the foot and underwent CT due to further examination for diagnosis. The disorders of the patients included in the study may influence the results. Nevertheless, the findings of this study suggest the possible clinical application of the simple footprint assessment board to detect flatfoot.

## Conclusions

The findings of this study suggest that a simple footprint assessment board can be potentially useful to aid the detection of flatfoot for all patients without need for specialized training. Further studies with a larger sample size and greater variation of comparative radiological indices should be conducted to validate the simple footprint assessment board as a standard procedure for the diagnosis of flatfoot.

## Supplementary Information

**Additional file 1.** Results of the age-stratified survey

## Data Availability

The datasets generated and/or analysed during the current study are available in the UMIN repository. UMIN000042719.

## Abbreviations

CT: Computer tomography
ICC: Intraclass correlation coefficient
