# Supplementary material for: Validity of a simple footprint assessment board for diagnosing the severity of flatfoot: a prospective cohort study
Source: BMC Musculoskelet Disord. 2021 Mar 18;22:285. doi: 10.1186/s12891-021-04154-3 (PMC7977274; doi:10.1186/s12891-021-04154-3)
Supplement: Supplementary file 1 — Additional file 1. Results of the age-stratified survey [file 12891_2021_4154_MOESM1_ESM.docx]

**Additional file 1. Results of the age-stratified survey**

**Validity of a simple footprint assessment board for diagnosing the severity of flatfoot: a prospective cohort study**

Seikai Toyooka, M.D.^1^, Naoya Shimazaki, M.D.^2^, Youichi Yasui, M.D.^1^, Shuji Ando, Ph.D. ^3^, Yasuaki Saho, Ph.D. ^4^, Takumi Nakagawa, M.D.^1^, Hirotaka Kawano, M.D.^1^, and Wataru Miyamoto, M.D.^1^

1. Department of Orthopaedic Surgery, Teikyo University School of Medicine, Tokyo, Japan
2. Department of Orthopaedic Surgery, Shimazaki Hospital, Ibaraki, Japan
3. Department of Information Engineering, Tokyo University of Science, Tokyo, Japan
4. Faculty of Medical Technology, Teikyo University Institute of Sports Science and Medicine

**Young Patients**

**A: Navicular index**

Regression analysis with the value of the simple footprint assessment board as a dummy variable

| Score of arch check board | n | Mean navicular index | Regression factor | Standard error | t-value | *P*-value | Coefficient of determination | Adjusted coefficient of determination |
| --- | --- | --- | --- | --- | --- | --- | --- | --- |
| (Intercept) |  |  | 4.93 | 0.5288 | 9.323 | 0.000 | 0.689 | 0.494 |
| Score 1 (Reference) | 1 | 4.9 |  |  |  |  |  |  |
| Score 1.5 | 4 | 5.2 | 0.230 | 0.591 | 0.389 | 0.707 |  |  |
| Score 2 | 4 | 3.9 | -1.073 | 0.591 | -1.814 | 0.107 |  |  |
| Score 2.5 | 3 | 4.0 | -0.930 | 0.611 | -1.523 | 0.166 |  |  |
| Score 3 | 1 | 3.9 | -1.070 | 0.748 | -1.431 | 0.190 |  |  |
| Score 3.5 | 1 | 3.7 | -1.230 | 0.748 | -1.645 | 0.139 |  |  |

Score of arch check board (explanatory variables: x）

Navicular index (objective variables: y)

Regression analysis with the value of the simple footprint assessment board as a continuous variable

| Score of arch check board | Regression factor | Standard error | t-value | *P*-value | Coefficient of determination | Adjusted coefficient of determination |
| --- | --- | --- | --- | --- | --- | --- |
| (Intercept) | 5.781 | 0.532 | 10.878 | 0.000 | 0.407 | 0.358 |
| Score | -0.703 | 0.245 | -2.871 | 0.014 |  |  |

Score of arch check board (explanatory variables: x）

Navicular index (objective variables: y)，

**B:** **Tibiocalcaneal angle**

Regression analysis with the value of the simple footprint assessment board as a dummy variable

| Score of arch check board | n | Mean navicular index | Regression factor | Standard error | t-value | *P*-value | Coefficient of determination | Adjusted coefficient of determination |
| --- | --- | --- | --- | --- | --- | --- | --- | --- |
| (Intercept) |  |  | 20.000 | 1.250 | 16.001 | 0.000 | 0.872 | 0.765 |
| Score 1 (Reference) | 1 | 20.0 |  |  |  |  |  |  |
| Score 1.5 | 4 | 23.9 | -4.475 | 1.397 | -3.202 | 0.019 |  |  |
| Score 2 | 4 | 25.0 | -6.000 | 1.531 | -3.919 | 0.008 |  |  |
| Score 2.5 | 3 | 29.2 | -6.667 | 1.443 | -4.619 | 0.004 |  |  |
| Score 3 | 1 | 30.0 | -6.800 | 1.768 | -3.847 | 0.008 |  |  |
| Score 3.5 | 1 | 35.0 | -10.300 | 1.768 | -5.827 | 0.001 |  |  |

Score of arch check board (explanatory variables: x）

Tibiocalcaneal angle (objective variables: y)

Regression analysis with the value of the simple footprint assessment board as a continuous variable

| Score of arch check board | Regression factor | Standard error | t-value | *P*-value | Coefficient of determination | Adjusted coefficient of determination |
| --- | --- | --- | --- | --- | --- | --- |
| (Intercept) | 20.655 | 1.278 | 16.157 | 0.000 | 0.726 | 0.699 |
| Score | -2.994 | 0.582 | -5.149 | 0.000 |  |  |

Score of arch check board (explanatory variables: x）

Tibiocalcaneal angle (objective variables: y)，

C: Calcaneal inclination angle

Regression analysis with the value of the simple footprint assessment board as a dummy variable

| Score of arch check board | n | Mean navicular index | Regression factor | Standard error | t-value | *P*-value | Coefficient of determination | Adjusted coefficient of determination |
| --- | --- | --- | --- | --- | --- | --- | --- | --- |
| (Intercept) |  |  | 20.000 | 1.257 | 15.906 | 0.000 | 0.950 | 0.908 |
| Score 1 (Reference) | 1 | 20.0 | 3.580 | 2.269 | 1.578 | 0.128 |  |  |
| Score 1.5 | 4 | 15.5 | 8.020 | 2.269 | 3.534 | 0.002 |  |  |
| Score 2 | 4 | 14.0 | 8.667 | 2.215 | 3.913 | 0.001 |  |  |
| Score 2.5 | 3 | 13.3 | 13.000 | 2.269 | 5.729 | 0.000 |  |  |
| Score 3 | 1 | 13.2 | 15.167 | 2.215 | 6.849 | 0.000 |  |  |
| Score 3.5 | 1 | 9.7 | 20.500 | 3.322 | 6.171 | 0.000 |  |  |

Score of arch check board (explanatory variables: x）

Calcaneal inclination angle (objective variables: y)

Regression analysis with the value of the simple footprint assessment board as a continuous variable

| Score of arch check board | Regression factor | Standard error | t-value | *P*-value | Coefficient of determination | Adjusted coefficient of determination |
| --- | --- | --- | --- | --- | --- | --- |
| (Intercept) | 15.261 | 1.167 | 13.080 | 0.000 | 0.912 | 0.903 |
| Score | 5.399 | 0.531 | 10.170 | 0.000 |  |  |

Score of arch check board (explanatory variables: x）

Calcaneal inclination angle (objective variables: y)，

**Middle-Aged Patients**

**A: Navicular index**

Regression analysis with the value of the simple footprint assessment board as a dummy variable

| Score of arch check board | n | Mean navicular index | Regression factor | Standard error | t-value | *P*-value | Coefficient of determination | Adjusted coefficient of determination |
| --- | --- | --- | --- | --- | --- | --- | --- | --- |
| (Intercept) |  |  | 4.873 | 0.283 | 17.245 | 0.000 | 0.645 | 0.493 |
| Score 2 (Reference) | 4 | 4.9 |  |  |  |  |  |  |
| Score 2.5 | 2 | 4.7 | -0.193 | 0.489 | -0.393 | 0.706 |  |  |
| Score 3 | 4 | 3.9 | -1.013 | 0.400 | -2.534 | 0.039 |  |  |
| Score 4 | 1 | 3.0 | -1.873 | 0.632 | -2.964 | 0.021 |  |  |

Score of arch check board (explanatory variables: x）

Navicular index (objective variables: y)

Regression analysis with the value of the simple footprint assessment board as a continuous variable

| Score of arch check board | Regression factor | Standard error | t-value | *P*-value | Coefficient of determination | Adjusted coefficient of determination |
| --- | --- | --- | --- | --- | --- | --- |
| (Intercept) | 6.889 | 0.694 | 9.926 | 0.000 | 0.620 | 0.577 |
| Score | -0.982 | 0.257 | -3.829 | 0.004 |  |  |

Score of arch check board (explanatory variables: x）

Navicular index (objective variables: y)，

**B: Tibiocalcaneal angle**

Regression analysis with the value of the simple footprint assessment board as a dummy variable

| Score of arch check board | n | Mean navicular index | Regression factor | Standard error | t-value | *P*-value | Coefficient of determination | Adjusted coefficient of determination |
| --- | --- | --- | --- | --- | --- | --- | --- | --- |
| (Intercept) |  |  | 14.833 | 1.268 | 11.694 | 0.000 | 0.584 | 0.334 |
| Score 2 (Reference) | 4 | 14.8 |  |  |  |  |  |  |
| Score 2.5 | 2 | 13.3 | -1.533 | 2.537 | -0.604 | 0.572 |  |  |
| Score 3 | 4 | 11.4 | -3.408 | 1.678 | -2.031 | 0.098 |  |  |
| Score 4 | 1 | 9.0 | -5.833 | 2.537 | -2.299 | 0.070 |  |  |

Score of arch check board (explanatory variables: x）

Tibiocalcaneal angle (objective variables: y)

Regression analysis with the value of the simple footprint assessment board as a continuous variable

| Score of arch check board | Regression factor | Standard error | t-value | *P*-value | Coefficient of determination | Adjusted coefficient of determination |
| --- | --- | --- | --- | --- | --- | --- |
| (Intercept) | 20.845 | 2.776 | 7.510 | 0.000 | 0.576 | 0.516 |
| Score | -3.066 | 0.994 | -3.086 | 0.018 |  |  |

Score of arch check board (explanatory variables: x）

Tibiocalcaneal angle (objective variables: y)，

**C: Calcaneal inclination angle**

Regression analysis with the value of the simple footprint assessment board as a dummy variable

| Score of arch check board | n | Mean navicular index | Regression factor | Standard error | t-value | *P*-value | Coefficient of determination | Adjusted coefficient of determination |
| --- | --- | --- | --- | --- | --- | --- | --- | --- |
| (Intercept) |  |  | 23.000 | 1.592 | 14.450 | 0.000 | 0.820 | 0.712 |
| Score 2 (Reference) | 4 | 23.0 |  |  |  |  |  |  |
| Score 2.5 | 2 | 27.0 | 4.000 | 3.183 | 1.257 | 0.264 |  |  |
| Score 3 | 4 | 30.0 | 7.000 | 2.106 | 3.325 | 0.021 |  |  |
| Score 4 | 1 | 37.0 | 14.000 | 3.183 | 4.398 | 0.007 |  |  |

Score of arch check board (explanatory variables: x）

Calcaneal inclination angle (objective variables: y)

Regression analysis with the value of the simple footprint assessment board as a continuous variable

| Score of arch check board | Regression factor | Standard error | t-value | *P*-value | Coefficient of determination | Adjusted coefficient of determination |
| --- | --- | --- | --- | --- | --- | --- |
| (Intercept) | 15.261 | 1.167 | 13.080 | 0.000 | 0.912 | 0.903 |
| Score | 5.399 | 0.531 | 10.170 | 0.000 |  |  |

Score of arch check board (explanatory variables: x）

Calcaneal inclination angle (objective variables: y)，

**Older Patients**

**A: Navicular index**

Regression analysis with the value of the simple footprint assessment board as a dummy variable

| Score of arch check board | n | Mean navicular index | Regression factor | Standard error | t-value | *P*-value | Coefficient of determination | Adjusted coefficient of determination |
| --- | --- | --- | --- | --- | --- | --- | --- | --- |
| (Intercept) |  |  | 6.370 | 0.576 | 11.057 | 0.000 | 0.694 | 0.311 |
| Score 0.5 (Reference) | 2 | 6.4 |  |  |  |  |  |  |
| Score 1 | 4 | 6.3 | -0.118 | 0.706 | -0.167 | 0.876 |  |  |
| Score 1.5 | 1 | 5.2 | -1.160 | 0.998 | -1.162 | 0.310 |  |  |
| Score 2 | 1 | 4.6 | -1.730 | 0.998 | -1.734 | 0.158 |  |  |
| Score 2.5 | 1 | 5.4 | -0.940 | 0.998 | -0.942 | 0.400 |  |  |
| Score 3 | 1 | 4.1 | -2.240 | 0.998 | -2.245 | 0.088 |  |  |

Score of arch check board (explanatory variables: x）

Navicular index (objective variables: y)

Regression analysis with the value of the simple footprint assessment board as a continuous variable

| Score of arch check board | Regression factor | Standard error | t-value | *P*-value | Coefficient of determination | Adjusted coefficient of determination |
| --- | --- | --- | --- | --- | --- | --- |
| (Intercept) | 6.938 | 0.439 | 15.791 | 0.000 | 0.562 | 0.507 |
| Score | -0.873 | 0.273 | -3.202 | 0.013 |  |  |

Score of arch check board (explanatory variables: x）

Navicular index (objective variables: y)

**B: Tibiocalcaneal angle**

Regression analysis with the value of the simple footprint assessment board as a dummy variable

| Score of arch check board | n | Mean navicular index | Regression factor | Standard error | t-value | *P*-value | Coefficient of determination | Adjusted coefficient of determination |
| --- | --- | --- | --- | --- | --- | --- | --- | --- |
| (Intercept) |  |  | 26.000 | 1.547 | 16.806 | 0.000 | 0.896 | 0.766 |
| Score 0.5 (Reference) | 2 | 26.0 |  |  |  |  |  |  |
| Score 1 | 4 | 20.0 | -5.925 | 1.895 | -3.127 | 0.035 |  |  |
| Score 1.5 | 1 | 15.7 | -10.300 | 2.680 | -3.844 | 0.018 |  |  |
| Score 2 | 1 | 18.0 | -8.000 | 2.680 | -2.986 | 0.041 |  |  |
| Score 2.5 | 1 | 14.1 | -11.900 | 2.680 | -4.441 | 0.011 |  |  |
| Score 3 | 1 | 13.7 | -12.300 | 2.680 | -4.590 | 0.010 |  |  |

Score of arch check board (explanatory variables: x）

Tibiocalcaneal angle (objective variables: y)

Regression analysis with the value of the simple footprint assessment board as a continuous variable

| Score of arch check board | Regression factor | Standard error | t-value | *P*-value | Coefficient of determination | Adjusted coefficient of determination |
| --- | --- | --- | --- | --- | --- | --- |
| (Intercept) | 25.750 | 1.622 | 15.874 | 0.000 | 0.719 | 0.684 |
| Score | -4.550 | 1.006 | -4.523 | 0.002 |  |  |

Score of arch check board (explanatory variables: x）

Tibiocalcaneal angle (objective variables: y)

**C: Calcaneal inclination angle**

Regression analysis with the value of the simple footprint assessment board as a dummy variable

| Score of arch check board | n | Mean navicular index | Regression factor | Standard error | t-value | *P*-value | Coefficient of determination | Adjusted coefficient of determination |
| --- | --- | --- | --- | --- | --- | --- | --- | --- |
| (Intercept) |  |  | 14.500 | 1.937 | 7.485 | 0.002 | 0.833 | 0.624 |
| Score 0.5 (Reference) | 2 | 14.5 |  |  |  |  |  |  |
| Score 1 | 4 | 17.6 | 3.100 | 2.372 | 1.307 | 0.261 |  |  |
| Score 1.5 | 1 | 17.0 | 2.500 | 3.355 | 0.745 | 0.498 |  |  |
| Score 2 | 1 | 20.0 | 5.500 | 3.355 | 1.639 | 0.177 |  |  |
| Score 2.5 | 1 | 14.1 | 8.500 | 3.355 | 2.533 | 0.064 |  |  |
| Score 3 | 1 | 13.7 | 13.500 | 3.355 | 4.024 | 0.016 |  |  |

Score of arch check board (explanatory variables: x）

Calcaneal inclination angle (objective variables: y)

Regression analysis with the value of the simple footprint assessment board as a continuous variable

| Score of arch check board | Regression factor | Standard error | t-value | *P*-value | Coefficient of determination | Adjusted coefficient of determination |
| --- | --- | --- | --- | --- | --- | --- |
| (Intercept) | 12.278 | 1.484 | 8.276 | 0.000 | 0.759 | 0.729 |
| Score | 4.616 | 0.920 | 5.017 | 0.001 |  |  |

Score of arch check board (explanatory variables: x）

Calcaneal inclination angle (objective variables: y)
